# Improvement of Radiotherapy-Induced Lacrimal Gland Injury by Induced Pluripotent Stem Cell-Derived Conditioned Medium via MDK and Inhibition of the p38/JNK Pathway

**DOI:** 10.3390/ijms151018407

**Published:** 2014-10-13

**Authors:** Yanqing Zhang, Chenliang Deng, Jiang Qian, Mingui Zhang, Xiaofeng Li

**Affiliations:** 1 Department of Ophthalmology, Eye and ENT Hospital of Fudan University, Shanghai 200031, China; E-Mails: rtpcr@163.com (J.Q.); dacier@126.com (M.Z.); googqq@126.com (X.L.); 2 Department of Plastic Surgery, Shanghai Jiaotong University Affiliated Sixth People’s Hospital, Shanghai 200031, China; E-Mail: alcee@126.com

**Keywords:** radiotherapy, P38, lacrimal gland, induced pluripotent stem cell

## Abstract

Radiation therapy is the most widely used and effective treatment for orbital tumors, but it causes dry eye due to lacrimal gland damage. Induced pluripotent stem cell-derived conditioned medium (iPSC-CM) has been shown to rescue different types of tissue damage. The present study investigated the mechanism of the potential radioprotective effect of IPS cell-derived conditioned medium (iPSC-CM) on gamma-irradiation-induced lacrimal gland injury (RILI) in experimental mice. In this study, we found that iPSC-CM ameliorated RILI. iPSC-CM markedly decreased radiotherapy induced inflammatory processes, predominantly through suppressing p38/JNK signaling. Further signaling pathway analyses indicated that iPSC-CM could suppress Akt (Protein Kinase B, PKB) phosphorylation. High levels of midkine (MDK) were also found in iPSC-CM and could be involved in lacrimal gland regeneration by promoting cell migration and proliferation. Thus, our study indicates that inhibiting the p38/JNK pathway or increasing the MDK level might be a therapeutic target for radiation-induced lacrimal gland injury.

## 1. Introduction

Radiotherapy-induced lacrimal gland injury (RILGI) is a major clinical concern for patients receiving malignant tumor radiotherapy [1,2,3]. Radiation damages lacrimal gland cells, resulting in cellular degeneration, necrosis and apoptosis [4]; therefore, tear secretion is impaired, and xerophthalmia is induced [1,2,5]. RILGI tissue has also been shown to elicit the production of inflammatory mediators, which recruit inflammatory cells [6]. However, satisfactory treatment strategies for this clinical problem are still lacking because of a poor understanding of this complex process. Therefore, exploring the underlying mechanisms for this process might help to identify new therapeutic targets. Lacrimal gland epithelial (LGE) cells are primary lacrimal gland cells [7] that are firmly implicated in lacrimal gland physiological homeostasis and pathology, including radiotherapy-induced lacrimal gland injury. LGE cells are necessary for tear secretion, and their proliferation and migration are crucial for lacrimal gland repair [8,9]. Bone marrow mesenchymal stem cells demonstrate promising improvement of the re-epithelization process and a reduction in T-cell infiltration and proliferation in a rat model of radiation [10]. Studies indicate that mesenchymal stem cells can effectively contribute to lacrimal gland epithelial cell repair after experimentally induced inflammation injury [8,9]. Recently, the treatment efficacy of iPS cell-derived conditioned medium (iPSC-CM) in restoring lung epithelial structural damage and suppressing neutrophil infiltration has been demonstrated in ventilator-induced lung injury [11]. However, the stem cell therapy-based biomolecular mechanisms that improve RILGI inflammation remain unknown.

The p38 mitogen-activated protein kinase (p38) pathway can be activated in cells and tissues in response to extracellular stimuli such as osmotic shock, hypoxia and gamma-irradiation injury [12,13]. Studies have shown that p38 plays a critical role in regulating cell survival and regeneration following exposure to irradiation. Furthermore, inhibition of p38 can promote *ex vivo* hematopoietic stem cell expansion and attenuate hematopoietic cell senescence induced by irradiation [13,14]. In the absence of MKP-1, p38-induced AKT activity anticipates the acquisition of the anti-inflammatory gene program and final cytokine silencing in macrophages, resulting in impaired tissue healing. Such defects were reversed by temporally controlling p38 inhibition [15].

However, the possible protective role of iPSC-CM and its underlying mechanisms, including the p38 pathway, in RILGI remain unknown. In the present study, we helped elucidate whether iPSC-CM could rescue RILGI via modulating the p38/JNK signal pathway and inflammatory response. Using western blotting, we identified candidate secreted proteins involved in the efficacy of iPSC-CM-mediated repair. Our findings may provide effective iPSC-based adjunctive therapies for lacrimal gland injury using malignant orbital tumor radiotherapy.

## 2. Results and Discussion

### 2.1. Results

#### 2.1.1. Improvement of a RILI Mouse Model by iPSC-CM

**Figure 1:**
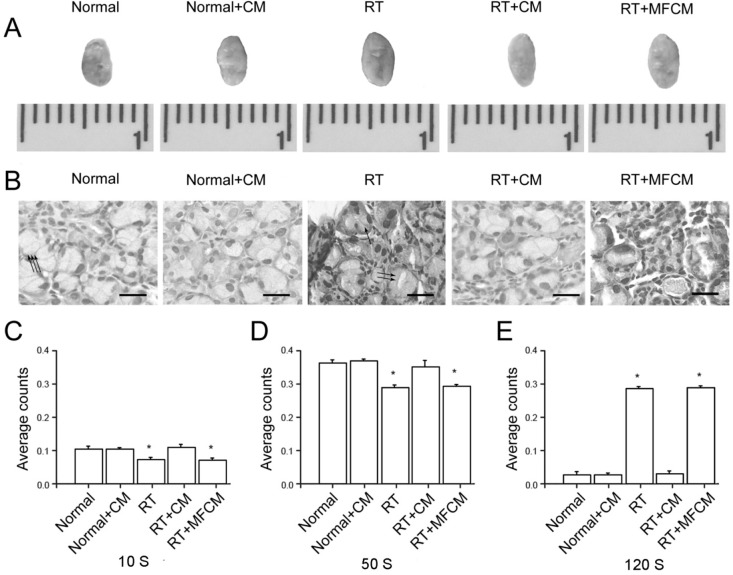
Radiotherapy-impaired lacrimal secretion and induced lacrimal gland injury *in vivo*. (**A**) Gross observation indicating normal and radiotherapy-treated lacrimal glands and the restorative effect of iPSC-CM or MFCM (mouse fibroblasts-derived conditioned medium) on irradiated lacrimal glands; (**B**) HE staining of irradiated lacrimal glands. Scale bar = 25 µm. One arrow means inflammatory cells, two arrows means the apoptotic acinar cells and three arrows means normal acinar cells; (**C–E**) Scintigraphic assessment of lacrimal gland secretion function of normal, normal + CM, RT, RT + CM, or RT + MFCM (CM: iPSC-CM, RT: radiotherapy, MFCM: mouse fibroblasts conditioned medium); *N* = 5. The values are the means ± SD. * *p* < 0.01.

**Figure 2:**
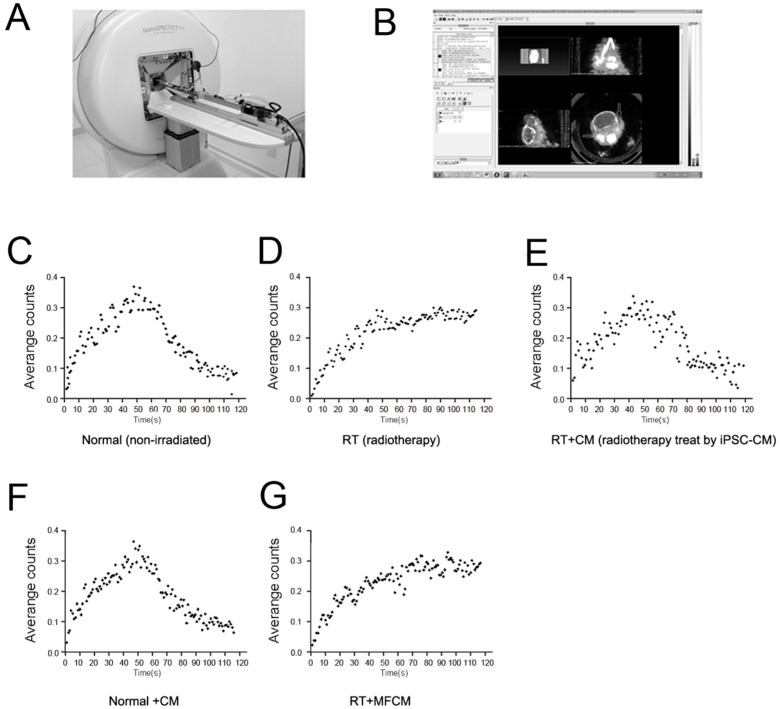
Supplementary figure. (**A**) The mice underwent sequential scintigraphy in a prone position with frontal projection of the head using a four-head camera; (**B**) Scintigraphic data analyzed by software; (**C–G**) Time–activity curve of 99 mTc pertechnetate in the major lacrimal glands of mice in the five groups.

The RILI animal models were established, gross observation were exert; histological and structural changes were observed using HE staining microscopy, and lacrimal gland scintigraphic evaluation was performed for the mouse model and normal animal groups (Figure 1C–E). The lacrimal gland of iPSC-CM-treated RILI and normal animal groups displayed a pattern of hemorrhaging, severe congestion and enlargement due to edema (Figure 1A). The lacrimal gland tissue showed tubulo-acinar structure of the inferior lacrimal gland, with a cubic, regular shape for the acinar cells and basally located nuclei (Figure 1B). However, secretory retention was observed in most acinar and tubular cells in the RILI model. Furthermore, scattered vasculopathy and an increase in the number of aberrant nuclei in apoptotic acinar cells were observed, along with extracellular edema and increased congestion of the interlobular blood vessels (Figure 1B). For the iPSC-CM-treated RILI and normal animal groups, the time-activity curve had a parabolic shape (Figure 2C,E). For the RILI animal model, secretary retention or obstruction happened due to the damage of lacrimal gland cell membrane by irradiation, the ejection function of the damaged lacrimal glands was destroyed, and the tracer accumulated. The time–activity curve showed an ascending tendency (Figure 2D). These results confirmed that, in the RILI animal model, radiation substantially damaged lacrimal gland function and structure, and iPSC-CM delivery improved the RILI mouse model.

#### 2.1.2. iPSC-CM Suppressed the RILI-Associated Inflammatory Response 

**Figure 3:**
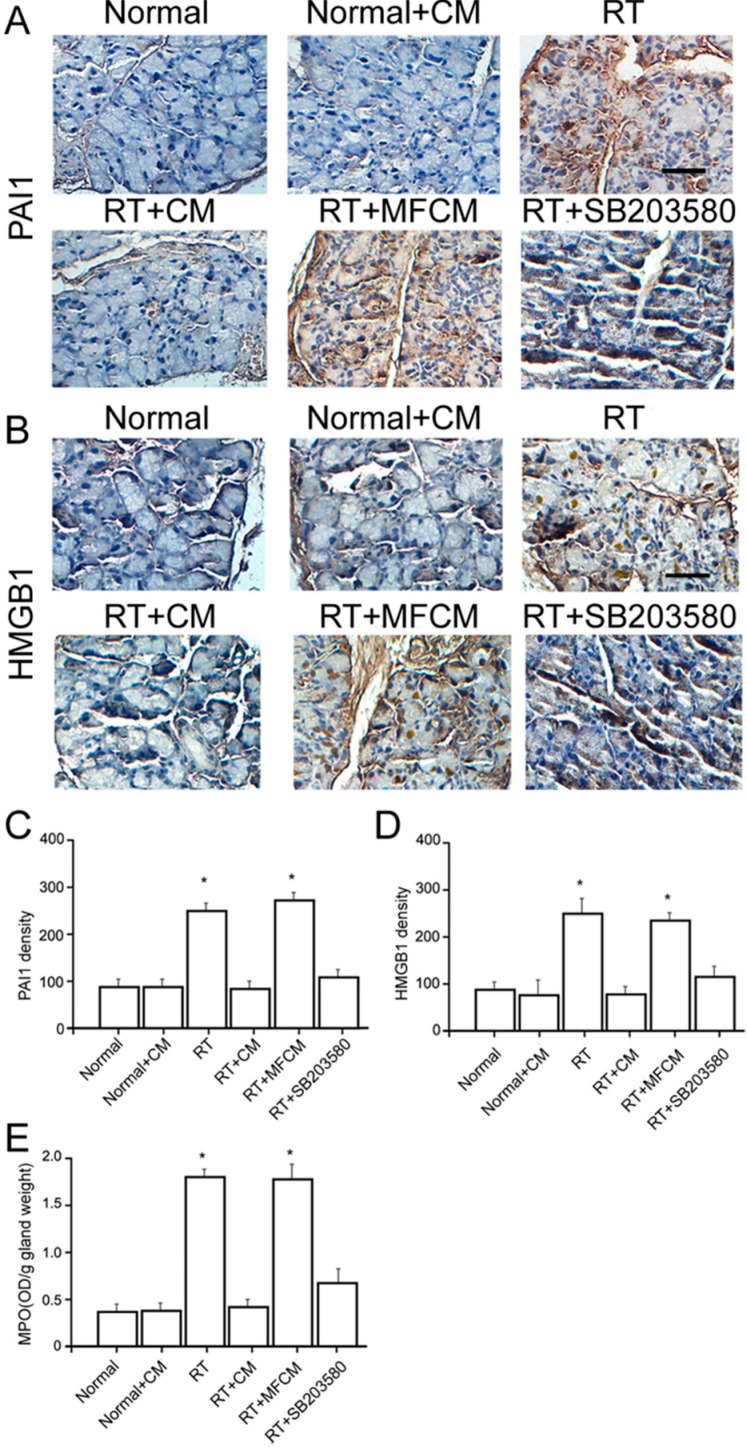
iPSC-CM suppressed the RILI-associated inﬂammatory response. (**A**) Immunohistochemical staining for PAI1 in iPSC-CM treated RILI and normal mouse lacrimal glands. Scale bar = 25 µm; (**B**) Immunohistochemical staining for HMGB1 in iPSC-CM treated RILI and normal mouse lacrimal glands. Scale bar = 25 µm; (**C,D**) Quantiﬁcation of the mean density of immunohistochemical staining of these sections (CM: iPSC-CM, RT: radiotherapy, MFCM: mouse fibroblasts conditioned medium). *N* = 5. Values are means ± SEM. * *p* < 0.01; (E) Neutrophils migrated into the injured gland sites revealed by the neutrophil counts and myeloperoxidase (MPO) assay. * *p* < 0.01.

We then examined whether iPSC-CM led to structural recovery in this RILI model. Histological examination revealed that radiotherapy led to congestion, hemorrhaging and neutrophil infiltration, which were largely rescued by the administration of iPSC-CM (Figure 1B). Scintigraphic evaluation confirmed the severe radiotherapy-induced damage and the therapeutic potential of iPSC-CM (Figure 1C–E). The neutrophil counts and myeloperoxidase (MPO) assay revealed that neutrophils migrated into the injured gland sites in the mice after radiotherapy, unlike in the non-radiotherapy mice (Figure 3E). Meanwhile, the HMGB1 and PAI-1 protein levels were elevated in response to RILI (Figure 3C,D), indicating an upregulation of chemoattractants for neutrophils in this model. Significantly, iPSC-CM ameliorated neutrophil migration and elevated the HMGB1 and PAI-1 protein levels (Figure 3C,D). These data demonstrate that iPSC-CM attenuates neutrophil infiltration and inflammatory responses in RILI.

#### 2.1.3. Ultramicrostructural Restoration by iPSC-CM

**Figure 4:**
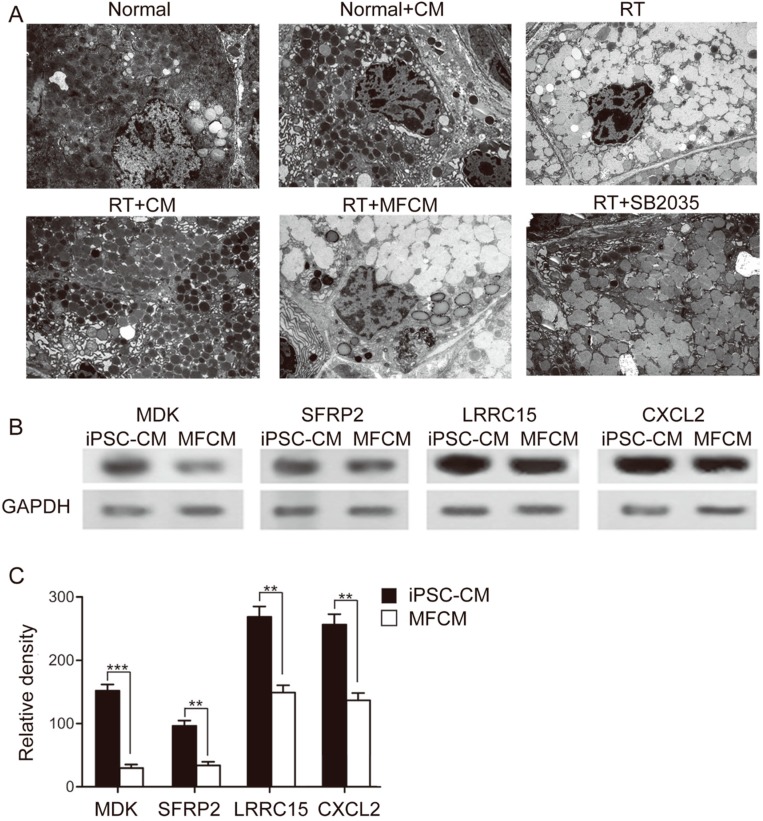
Ultramicrostructural restoration by iPSC-CM. (**A**) A TEM image revealing the lacrimal gland ultramicrostructure in normal, normal + CM, RT, RT + CM, or RT + MFCM (normal + CM: non irradiated after iPSC-CM treatment, RT: radiotherapy, RT + CM: radiotherapy after iPSC-CM treatment, RT + MFCM: radiotherapy after mouse fibroblasts conditioned medium treatment); *N* = 5; (**B**) Western blot of the protein level of MDK, SFRP2, CXCL2 and LRRC15 in iPSC-CM and MFCM (MFCM: mouse fibroblasts conditioned medium); (**C**) Quantification of the relative density of western blot results. *N* = 5. Values are means ± SEM. ** *p* < 0.01, *** *p* < 0.001.

Transmission electron microscopy (TEM) showed that administration of radiotherapy led to an intracellular retention of secretory granula with subsequent displacement of the acinar nuclei in lacrimal glands 3 days after radiation, indicating acute injury of the lacrimal gland ultramicrostructure in the RILI model (Figure 4A). In the parallel experimental group of the RILI model treated with iPSC-CM, apoptotic acinar nuclei were observed, and partial remission that included reduction of secretory retention was noticed. Administration of iPSC-CM consistently restored the lacrimal gland ultramicrostructure in the recipients, similar to the treatment effect of p38 inhibition (Figure 4A). The protein level of MDK, SFRP2, CXCL2 and LRRC15 was obviously higher in iPSC-CM than that in fibroblast-conditioned medium (Figure 4B,C), as shown by western blotting, which suggested that one or some were most likely involved in the efficacy of iPSC-CM (Figure 4B,C).

#### 2.1.4. Recombinant MDK Promotes LGE Cell Migration and Proliferation

**Figure 5:**
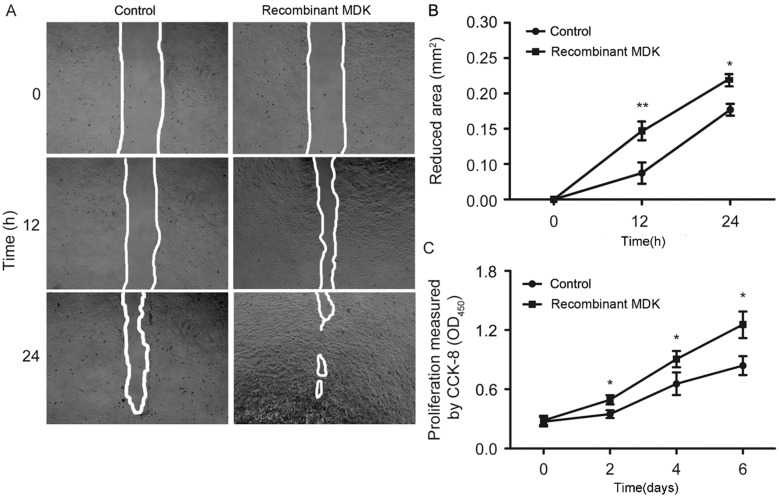
Recombinant MDK (Midkine) promotes LGE (Lacrimal gland epithelial) cell migration and proliferation. (**A**) Images of LGE cell scratch-wound healing assays. Scale bar = 250 µm; (**B**) Quantification of the reduced area; *N* = 5. The values are the means ± SEM. * *p* < 0.05, ** *p* < 0.01; (**C**) *In vitro* proliferation assay for LGE cells treated with MDK; *N* = 5. The values are the means ± SEM. * *p* < 0.05.

LGE cells are the primary cell type in the lacrimal gland [7], and the normal motility and proliferation of LGE cells is pivotal for lacrimal gland repair after local radiotherapy. Accumulating evidence has demonstrated that MDK has positive roles in cell proliferation and migration [16,17]. To explore the functional roles of MDK in radiation-induced lacrimal gland injury, we observed the effects of recombinant MDK protein on the migration and proliferation of isolated primary LGE cells. Our results revealed that after treatment with recombinant MDK protein, LGE cell migration (Figure 5A,B) and proliferation (Figure 5C) were significantly promoted *in vitro*. This finding indicated that the upregulation of MDK might promote the recovery that is potentially induced by LGE cell migration and proliferation.

#### 2.1.5. iPSCM Inhibits JNK, p38 and Akt Phosphorylation in LGE Cells

**Figure 6:**
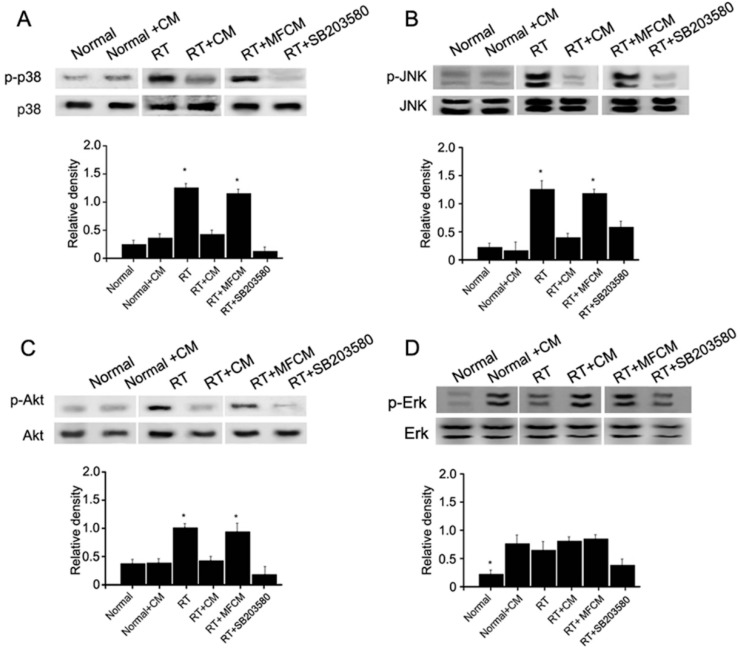
iPSC-CM suppresses the p38/JNK pathway. (**A**) Western blot (**upper**) and quantiﬁcation (**lower**) of p38, JNK (**B**), Akt (**C**) and Erk (**D**) phosphorylation from the irradiation injury lacrimal gland showing the effects of administering MFCM, p38 inhibitor (SB203580, a p38 MAPK inhibitor with IC50 of 0.3–0.5 μM.) or iPSC-CM on p38 phosphorylation in irradiation injury lacrimal glands. Lacrimal injury was induced in mice receiving radiotherapy. Data shown are the mean ± SD of five independent experiments. In (**A**), (**B**) and (**C**) * *p*<0.05 *vs.* normal, normal + CM, RT + CM, or RT + SB203580; In (**D**) * *p* < 0.05 *vs.* normal + CM, RT, RT + CM, RT + MFCM, or RT + SB203580 (normal + CM: non-irradiated after iPSC-CM treatment, RT: radiotherapy, RT + CM: radiotherapy after iPSC-CM treatment, RT + MFCM: radiotherapy after mouse fibroblasts conditioned medium treatment); *N* = 5.

MDK has been reported to be associated with Akt signaling and MAPK stress signaling, with involvement of p38 and JNK. To study whether these kinases are affected by the overexpression of MDK in LGE cells, we conducted western blot assays of these signaling kinases. We found that Akt, p38 and JNK phosphorylation could be significantly promoted by MDK overexpression (Figure 6A–C), but Erk1/2 was not obviously affected (Figure 6D).

### 2.2. Discussion

RILGI is characterized by inflammatory cell infiltration, apoptotic acinar cells, and extracellular edema at early stages [18], and tissue fibrosis [19] and atrophy at late stages, which ultimately lead to impaired tear secretion [3]. Mesenchymal stem cells (MSCs) were recently demonstrated to protect against severe inflammatory-induced murine lacrimal gland injury [8]. Lacrimal gland acinar cells are lost during inflammatory injury mesenchymal stem cells’ generation to form acinar and ductal epithelial cells during the epithelial-mesenchymal transition [8,9]. MSCs were also demonstrated to protect against inflammatory injury in a light-induced retinal degeneration rat model [20] and ischemia/reperfusion injury of mouse cardiac infarction [21]. Transplantation of mesenchymal stem cells (MSCs) at early and later stages after local exposure of rats to 140 Gy 90Sr/90Y beta radiation was found to stimulate recovery of damaged skin [22]. Human neural stem cell transplantation ameliorates radiation-induced cognitive dysfunction [23]. iPSC or iPSC-CM is beneficial for recovery from the effects of endotoxin-induced acute lung injury in mice [24]. Another study further indicated that iPSC and iPSC-CM both attenuated inflammatory injury through macrophage inflammatory protein-2, urokinase plasminogen activator (uPA), angiopoietin-1, tissue inhibitor of metalloproteinase (TIMP)-1 and TIMP-4, which all contribute to the decrease in inflammation [11]. iPSCs exert immunomodulatory effects, as observed by the prevention of allergic airway inflammation [25]. However, the mechanisms and mediators of iPSC- or iPSC-CM-dependent treatment in RILGI are still unclear and must be evaluated in preclinical studies.

In this study, we found that iPSC-CM reduced radiotherapy-induced lacrimal gland injury by decreasing congestion, edema and neutrophil infiltration in lacrimal glands. iPSC-CM also improved tear secretion activity in the RILI model. This report highlights the therapeutic potential of iPSC-CM in the treatment of xerophthalmia in RILI.

It was demonstrated in our previous study that Akt, p38 and JNK phosphorylation could be significantly promoted in a RILGI mouse model [19]. The phosphorylation of JNK was markedly increased upon irradiation-induced damage in rat submandibular glands [26]. The activation of the p38 pathway or phosphorylation of p38 has been demonstrated to predominantly contribute to irradiation-induced damage and bone marrow suppression [15,27,28,29]. Inhibition of p38 mitogen-activated protein kinase can promote *in vitro* hematopoietic stem cell proliferation, therefore, improving tissue repair [14]. While inhibition of the p38 pathway markedly attenuates irradiation-induced damage and increases tissue repair [27]. Our data indicate that RILGI induces the expression of p38 and JNK, which can be suppressed by iPSC-CM, and reduces neutrophil infiltration. This mechanism, which involves suppression of the p38 pathway by iPSCs/iPSC-CM, was further validated using SB203580 treatment.

Similar effects of iPSC-CM on p38 inhibition were observed in extracellular edema and tear production. These data validated the crucial role of the p38 pathway in the pathogenesis of RILGI, and blocking p38 signaling using iPSC-CM potentially restored a variety of lacrimal gland epithelium abnormalities in RILGI.

Midkine (MDK) was first characterized during the early differentiation stage of embryonic life [30]. MDK is a heparin-binding growth factor that is reported to promote the proliferation, differentiation, survival, adhesion and migration of cells [31,32,33,34]. Deletion of MDK results in a delay in regeneration, preceded by decelerated migration of macrophages to the damaged area after skeletal muscle injury [35]. In this study, recombinant MDK promoted the proliferation and migration of LGE cells. In addition to MDK, we found that other cytokines and secretary factors, including SFRP2 (secreted frizzled-related protein 2, modulator of Wnt signaling, regulating cell growth and differentiation in specific cell types), CXCL2 (chemokine (C-X-C motif) ligand 2, produced by activated monocytes and neutrophils and expressed at sites of inflammation) and LRRC15 (leucine rich repeat containing 15, a 581 amino acid protein that contains 15 LRR repeats and is involved in cell-cell and/or -extracellular matrix interactions), are highly expressed in iPSC-CM. These cytokines may also contribute to the decrease in inflammation and increase in lacrimal gland repair.

## 3. Experimental Section

### 3.1. Animal Preparation and Irradiation

Ten healthy, 8-week-old female C57BL/6 mice were used for the study. The mice underwent initial lacrimal gland scintigraphy. The first group (*n* = 5) was irradiated with a dose of 15 Gy under general anesthesia using a combination of 3 mg/kg (*S*)-ketamine-hydrochloride (Ketanest-S^®^, Parke-Davis, Hoofddorp, The Netherlands) and 0.1 mg/kg xylazine-hydrochloride (Rompun^®^, Bayer, Germany). Three days after irradiation, scintigraphy was performed for a second time, with subsequent excision of the left-side inferior lacrimal gland for histological examination. Seven days later, the same procedure was performed with the removal of the contralateral lacrimal gland. The second group (*n* = 5) was sham-treated but kept unirradiated as control glandular tissue.

### 3.2. Ethics Statement

The experimental procedures were approved by the Fudan University Animal Care and Use Committee, and all animals were housed under standard conditions according to institution-approved guidelines as previously described [19].

### 3.3. Surgical Harvesting of the Inferior Lacrimal Gland

The inferior lacrimal gland was surgically exposed and excised, and the harvested glands were divided into two parts. One part was fixed immediately with neutral phosphate-buffered with 4% formalin, and the other was fixed for transmission electron microscopy (CM 120, Phillips, Amsterdam, The Netherlands).

### 3.4. Lacrimal Gland Scintigraphy

After intravenous administration of 3.7 MBq (1 mCi = 37 MBq, 100 µCi = 3.7 MBq) Na^99m^TcO4 as a tracer, the mice underwent sequential scintigraphy in a prone position with frontal projection of the head using a four-head camera (Picker CX 250 compact, LEHR collimator and field-of-view of 25 cm; Nano SPECT/CT Plus, Bioscan Corporation) (Figure 2A,B). Time–activity curves were additionally registered (Figure 2C–G) and analyzed (Figure 1C–E).

### 3.5. Mouse Embryonic Fibroblasts iPSCs and Conditioned Medium

Murine-iPSCs were generated from non-reprogrammed MEFs that were derived from C57BL/6 mice. The iPSCs were reprogrammed by the transduction of retroviral vectors encoding three transcription factors, Oct-4, Sox2, and Klf4, as described previously [36]. The conditioned medium (200 μL) from iPSCs (iPSC-CM) or mouse fibroblasts (MFCM) (200 μL) were injected through tail vein 1 h before irradiation based on previous *in vivo* studies [37,38,39].

### 3.6. Pharmacological Inhibitor

A p38 inhibitor (5 mg/g; SB203580; Sigma Aldrich, St Louis, MO, USA) was given intraperitoneally 1 h before irradiation based on dose-response studies that showed that 5 mg/g inhibited p38 MAPK activity [40,41].

### 3.7. Transmission Electron Microscopy

After embedding in Araldite, semi-thin sections were stained with methylene blue to visualize the epithelial cells. Ultrathin sections were cut and stained with lead citrate and examined using a transmission electron microscope (CM 120, Phillips, Amsterdam, The Netherlands).

### 3.8. Immunohistochemistry

Paraformaldehyde-fixed, paraffin-embedded mouse lacrimal gland sections (4 µm) were first incubated with a primary antibody against mouse PAI1 (Molecular Innovations, Inc., Southfield, MI, USA) and HMGB1 (R&D System Inc., Minneapolis, MN, USA) overnight at 4 °C and then incubated with the goat anti-mouse IgG secondary antibodies (Molecular Innovations, Inc., Southfield, MI, USA). The sections were developed with diaminobenzidine and counterstained with hematoxylin. Quantification of the staining intensity was conducted by two independent investigators.

### 3.9. Cell Isolation and Culture

Primary mouse LGE cell isolation and culture were performed as previously reported [17]. LGE cells at passages 2–6 were used for experiments.

### 3.10. Cell Proliferation Assay

Proliferation was determined using a standard CCK-8 kit (Dojindo, Kumamoto, Japan) according to the manufacturer’s instruction. LGE cells treated with MDK or control cells (4.0 × 10^4^ cells/mL) were seeded in 96-well plates (100 μL/well). The optical density (OD450) values were measured at days 0, 2, 4 and 6 using a micro plate reader (SpectraMax M5, Molecular Devices Corporation, Sunnyvale, CA, USA).

### 3.11. Scratch-Wound Healing Assay

LGE cells treated with MDK or control cells were seeded in 24-well plates (2.5 × 10^5^ cells/well) and cultured to confluency. Next, the monolayer was gently scratched across the center with a 10-µL pipette tip, and the gaps were photographed at 0, 12 and 24 h post-scratch using a live-cell imaging system (Olympus, Tokyo, Japan).

### 3.12. Western Blot Assay

Proteins in iPSC-CM were collected using gradient centrifugation. The primary antibodies utilized were against the proteins MDK, Akt, p-Akt, p38, p-p38, JNK, p-JNK, Erk and p-Erk. All antibodies were purchased from Cell Signaling Technology. Signals were detected using an Odyssey Infrared Imaging System (LI-COR, Lincoln, NE, USA) after incubation with IRDye 800 anti-rabbit (LI-COR, Lincoln, NE, USA) secondary antibodies. Quantification was conducted using the Image J software (NIH, Bethesda, ML, USA).

### 3.13. Statistical Analysis 

Statistical analyses were performed using a two-tailed Student’s *t* test and *p* < 0.05 was considered to be statistically significant.

## 4. Conclusions

Our results demonstrate a protective effect by iPSC-CM in radiotherapy-injured lacrimal glands. We showed using a mouse model that radiotherapy-induced lacrimal gland injury is associated with increased neutrophil influx and the production of p38, as well as overproduction of oxidative substances, which can be ameliorated by iPSC-CM. The mechanisms by which iPSC-CM suppress these RILGI characteristics involved inhibition of the p38 pathway and MDK-dependent regulation. Therefore, intravenous delivery of iPSC-CM may serve as a potential advance in the management of RILGI. Further investigations of the paracrine and cytokine effects of iPSC-CM or iPSC-derivatives as therapeutic agents in different types of RILGI are needed.
